# Association of HIV Diversity and Survival in HIV-Infected Ugandan Infants

**DOI:** 10.1371/journal.pone.0018642

**Published:** 2011-04-14

**Authors:** Maria M. James, Lei Wang, Philippa Musoke, Deborah Donnell, Jessica Fogel, William I. Towler, Leila Khaki, Clemensia Nakabiito, J. Brooks Jackson, Susan H. Eshleman

**Affiliations:** 1 Johns Hopkins University School of Medicine, Baltimore, Maryland, United States of America; 2 Fred Hutchinson Cancer Research Center, Seattle, Washington, United States of America; 3 Makerere University-Johns Hopkins University Research Collaboration (MU-JHU), Baltimore, Maryland, United States of America, and Kampala, Uganda; 4 Makerere University School of Medicine, Kampala, Uganda; National AIDS Research Institute, India

## Abstract

**Background:**

The level of viral diversity in an HIV-infected individual can change during the course of HIV infection, reflecting mutagenesis during viral replication and selection of viral variants by immune and other selective pressures. Differences in the level of viral diversity in HIV-infected infants may reflect differences in viral dynamics, immune responses, or other factors that may also influence HIV disease progression. We used a novel high resolution melting (HRM) assay to measure HIV diversity in Ugandan infants and examined the relationship between diversity and survival through 5 years of age.

**Methods:**

Plasma samples were obtained from 31 HIV-infected infants (HIVNET 012 trial). The HRM assay was used to measure diversity in two regions in the *gag* gene (Gag1 and Gag2) and one region in the *pol* gene (Pol).

**Results:**

HRM scores in all three regions increased with age from 6–8 weeks to 12–18 months (for Gag1: P = 0.005; for Gag2: P = 0.006; for Pol: P = 0.016). Higher HRM scores at 6–8 weeks of age (scores above the 75^th^ percentile) were associated with an increased risk of death by 5 years of age (for Pol: P = 0.005; for Gag1/Gag2 (mean of two scores): P = 0.003; for Gag1/Gag2/Pol (mean of three scores): P = 0.002). We did not find an association between HRM scores and other clinical and laboratory variables.

**Conclusions:**

Genetic diversity in HIV *gag* and *pol* measured using the HRM assay was typically low near birth and increased over time. Higher HIV diversity in these regions at 6–8 weeks of age was associated with a significantly increased risk of death by 5 years of age.

## Introduction

In resource-limited countries, approximately half of all HIV-infected children who do not initiate antiretroviral therapy die by 2 years of age [1]. While early antiretroviral treatment dramatically decreases infant mortality [2], many resource-limited countries lack established programs for treatment of HIV-infected infants, and many infants in rural areas are not able to access care [3]. Maternal factors associated with mortality among HIV-infected infants include high HIV viral load, advanced HIV disease, early cessation of breastfeeding, and primary HIV infection [4], [5], [6], [7]. Infant factors include HIV infection before one month of age, low CD4 cell %, and high viral load [5], [6]. Viral characteristics, such as HIV subtype D [8], have also been associated with increased mortality of HIV-infected infants in some studies.

Several studies have found an association between higher HIV diversity (higher levels of genetic variation among HIV variants in the viral population) and more rapid HIV disease progression in adults [9], [10], [11], [12], [13]. In adults, higher HIV diversity prior to antiretroviral treatment was also associated with less effective virologic suppression following a strategic treatment interruption [14]. Viral dynamics and immune responses to HIV infection differ in infants, children, and adults with HIV infection. Those factors are likely to influence HIV diversity. Relatively little is known about the relationship between HIV diversity and disease progression in infants and young children. Previous studies reveal that most HIV-infected infants have genetically homogenous viral populations, indicating that one or a few HIV variants usually initiate infant infection [15]. HIV diversity generally increases in HIV-infected children over time [16], in response to immune and other selective pressures. In one study, sequence-based analysis of *env* in seven HIV-infected infants did not find an association between HIV diversity and disease progression [17]. Other studies (five to six infants each) found that greater diversification of *env* sequences over time was associated with slower disease progression [18], [19], [20].

To date, most studies of HIV diversity have been performed by analyzing sequences from individual HIV variants. The cost and complexity of those methods often limit the number of samples that can be analyzed, and therefore the scope of the studies performed. We recently developed a rapid assay for HIV diversity based on high resolution melting (HRM) of DNA duplexes [21], [22]. This HRM assay is simpler, less expensive, and requires less labor than traditional, sequence-based assays used to quantify genetic diversity; therefore, it can more easily be applied to larger sample sets. This assay uses a high resolution melting instrument (LightScanner, Idaho Technology, Inc., Salt Lake City, UT) to measure the range of temperatures at which DNA duplexes melt. Results from the HRM assay are expressed as a single number, the HRM score. In a reproducibility study, results obtained with the HRM assay were not significantly affected by differences in the HIV viral load of the plasma sample used for analysis (range: 2,000 to 50,000 copies/ml), the sample volume (100 vs. 500 ul), or the maximum number of HIV RNA copies used for amplification of DNA templates for HRM analysis (range 100 to 5,000 copies of HIV RNA). Furthermore, DNA templates prepared using replicate reverse transcription and amplification reactions had similar HRM scores (coefficient of variation: 0.064) [see File S1]. There was very little variation in HRM scores obtained when the same DNA templates were analyzed repeatedly over the course of a year (intra-class correlation coefficient: 94% [95% CI: 89%, 98%]) [21].

In a previous study, we cloned and sequenced HIV from nine of the infants described in this report (20 sequences from each infant sample, collected at 6–8 weeks of age) and their mothers (50 sequences for each maternal sample, collected near the time of delivery) [21]. The infants had relatively low HRM scores (median 4.3, range 4.2–5.3) and low sequence-based genetic diversity (median 0.31%, range 0.10–1.60%), while the mothers had a wider range of HRM scores (median 5.7, range 4.4–7.7) and higher sequence-based genetic diversity (median 3.57%, range 1.69–5.85%) [21]. Overall, HRM scores were significantly associated with sequence-based measures of HIV diversity, including genetic diversity, sequence complexity, and Shannon entropy [21]. In this study, we analyzed HIV diversity using the HRM assay, and used those data to evaluate the relationship between HIV diversity and disease progression in HIV-infected Ugandan infants.

## Methods

### Source of samples used for analysis

Plasma samples were obtained from Ugandan infants in the HIVNET 012 study (enrollment 1997–1999) [23], [24]. This sub-study included HIV-infected infants who received single dose nevirapine (sdNVP) for prevention of mother-to-child transmission, as well as infants who received no antiretroviral drugs for prophylaxis. None of the infants received any other antiretroviral drugs for treatment or prevention of mother-to-child transmission of HIV by 6–8 weeks of age. Plasma samples collected at 6–8 weeks of age were used for HRM analysis; 6–8 week samples were available for 25 of 37 infants in the sdNVP arm and from all six infants in the placebo arm who were HIV-infected by 6–8 weeks of age (total: 31 infants). HIV viral loads were obtained for 11 of the 31 6–8 week samples (median: 464,335 copies/ml; range: 44,454 to 7,993,300 copies/ml); all of those 11 infants were in the sdNVP arm of the HIVNET 012 trial. We used 100 ul of plasma to extract HIV RNA for HRM analysis and used 1/5^th^ of the extracted HIV RNA to amplify the DNA templates that were analyzed in the HRM assay. Therefore, among the 11 samples with viral load data, the number of HIV RNA copies used to prepare DNA templates for HRM analysis ranged from 889 to 159,866 copies. Samples from 9 of those 11 infants were analyzed in the HRM assay validation study described above [21].

Plasma samples collected at 12 and 18 months of age were also available for a subset of the 31 infants; those samples were also analyzed with the HRM assay. Those data were used to evaluate the relationship between HRM scores obtained at 6–8 weeks, 12 months, and 18 months of age. Infants were followed in the HIVNET 012 trial to 5 years of age. Clinical and laboratory data for these infants and their mothers was obtained from the HIVNET 012 trial database. Twenty-six (83.9%) of the 31 infants had viral load data obtained at 14 weeks of age; those data were used to adjust for HIV viral load when assessing the association between high HRM score and survival.

### Laboratory methods

HIV viral load and CD4 cell counts were determined in the HIVNET 012 trial. *In utero* HIV infection was defined as having a positive HIV culture or positive HIV RNA assay at birth.

HIV resistance testing was performed previously using the ViroSeq HIV Genotyping System (Celera, Alameda, CA), as described [25]. HIV subtyping was performed by phylogenetic analysis of HIV *pol* sequences, as described [26], with the following modifications: sequence alignments were generated using MegAlign (Lasergene 7, DNAStar, Madison, WI), and PHYLIP v3.66 was used for analysis.

**Figure 1:**
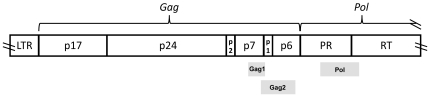
Regions analyzed in the HRM assay. The figure shows the regions of the HIV genome analyzed in the HRM assay (Gag1, Gag2, Pol). The HIV genome is represented by a non-shaded bar. The HRM amplicons are represented by shaded bars. LTR: long terminal repeat; PR: protease; RT: reverse transcriptase.

The HRM assay was performed as described [21]. Briefly, PCR products generated in the ViroSeq system were used as template DNA. In each reaction, the region of interest was amplified in a nested PCR reaction that included a fluorescent dye that was incorporated into the amplified DNA duplexes. The primer sequences and regions amplified are shown in Table 1 and Figure 1. The Gag1 amplicon includes a portion of the coding regions for *gag* p7 and *gag* p1. The Gag2 amplicon includes a portion of the coding regions for *gag* p7, *gag* p1, and *gag* p6. The Pol amplicon includes a portion of the coding regions for protease and reverse transcriptase. The LightScanner instrument was used to warm the samples over a melt range 68°C to 98°C, and to monitor the change in fluorescence that resulted as the DNA duplexes melted and the fluorescent dye was released; this step of the analysis takes approximately 10 minutes to perform. Data from the LightScanner instrument were used to produce a melting curve for each sample that displayed the change in fluorescence as a function of temperature [-d(fluorescence)/d(temperature) plotted against temperature]. Those curves were used to determine the number of degrees of temperature over which melting occurred (defined as the HRM score) [21]. Note that the actual melting temperatures for each sample (e.g., temperature at which melting begins, peak melting temperature, temperature at which melting is completed) are not considered in determining the HRM score. Each DNA sample was analyzed in duplicate; results were deemed acceptable if the HRM scores from the duplicate assays differed by <0.5.

The actual temperatures at which melting starts and stops for each region tested vary among viral populations from different HIV-infected individuals, and also vary among individual HIV variants (e.g., in cloned plasmids derived from individual viruses). The HRM assay was specifically designed not to take these temperatures into account, but instead to measure the number of degrees over which melting occurs. This is based on prior observations that homogeneous DNA duplexes melt over a fairly narrow temperature range, while more complex mixtures of DNA duplexes melt over a wider temperature range. The actual melting temperatures are not relevant to the level of genetic diversity, but instead reflect other features of the DNA, such as the specific DNA sequence, GC content, the presence of GC-rich domains, sequence length, etc.

**Table 1:** Use of the HRM assay to analyze different regions of the HIV genome.

Region analyzed	Gag1	Gag2	Pol
Corresponding region in HXB2*	1998 - 2096	2071–2278	2373–2597
Primer sequences			
Forward	5′-AAATTGCAGGGCCCCTAGGAA	5′-ACTGAGAGACAGGCTAATTTTTTAG	5′-AAATGGAAACCAAAAATGATAG
Reverse	5′-TTTCCCTAAAAAATTAGCCTGTCT	5′-GGTCGTTGCCAAAGAGTGATTTG	5′-CATTCCTGGCTTTAATTTTACTG
HRM amplicon size	99 bp	208 bp	225 bp
HRM scores for plasmids^a^ Median (range)	3.8 (3.8, 3.8)	3.4 (3.2, 3.8)	3.5 (3.4, 3.5)
HRM scores for 6–8 week old infants (n = 31)b,c Median (range)	4.7 (4.4, 7.3)	4.2 (3.6, 6.3)	4.1 (3.3, 6.7)
HRM scores for 12 month old infants (n = 17)^c^ Median (range)	5.2 (4.3, 7.1)	4.6 (3.8, 6.4)	4.3 (3.3, 5.9)
HRM scores for 18 month old infants (n = 15)^c^ Median (range)	5.1 (4.6, 6.3)	4.9 (4.2, 7.8)	4.4 (4.0, 5.9)

a Control reagents; two subtype A plasmids and two subtype D plasmids.

b One infant did not have an HRM result for the *Pol* region.

c These HRM scores can be compared to those obtained in an observational study of 79 children in Uganda [51] (median age 4.7 years, range 0.6–12.4 years). In that study, the median HRM score in the Gag2 region was 5.9 (range: 3.8–11.9).

HRM results were also obtained for control plasmids. Since plasmids are clonal (i.e., they have little or no genetic diversity), plasmid HRM scores serve as baseline values corresponding to the lowest HRM scores that are obtained for each region analyzed. Each of the plasmids used as a control in this study was derived from a different individual; for this reason, differences in the HRM scores for the plasmids tested are expected to be larger than the differences that one would expect when comparing the HRM scores from plasmids isolated from a single individual.

### Statistical Methods

Summary statistics (median and range for continuous variables; frequency distributions for categorical variables) were provided for clinical and laboratory variables; comparisons used Fisher's exact test and the exact Wilcoxon rank sum test. Mean HRM scores at three different HIV genomic regions in either plasmids or infant samples from 6–8 weeks were compared using ANOVA. Logistic regression modeled the probability of being in the high HRM group (HRM score above the 75^th^ percentile) and assessed the association between clinical and laboratory characteristics for HRM scores measured at 6–8 weeks, using Firth's penalized likelihood approach to avoid bias in parameter estimates caused by small sample size.

Log rank tests were used to compare Kaplan Meier survival curves of infants categorized into high vs. low HRM groups (above vs. below the 75^th^ percentile), based on the HRM score obtained from their 6–8 week samples; this was done separately for each region analyzed (Gag1, Gag2, Pol), for the mean HRM score for the two *gag* regions, and for the mean for all three HRM regions. The choice of breakpoint for the HRM scores was data driven, chosen amongst the 25^th^, 50^th^ and 75^th^ percentile as best discriminating the 5-year mortality outcome [see File S2]; the 75^th^ breakpoint values were 5.0 for Gag1, 4.8 for Gag2, and 4.3 for Pol. Infants were censored at their last follow-up visit in the five-year follow-up of the HIVNET 012 trial. One infant who initiated antiretroviral treatment during the study was censored at the time of treatment initiation.

A Cox proportional hazard model was used to assess the association between high HRM score and survival, while adjusting for viral load at 14 weeks of age (log_10_ scale). Linear mixed-effect models that account for potential correlations among repeated measures from the same subject were used to evaluate the relationship between age and HRM scores obtained at 6–8 weeks, 12 months, and 18 months. All statistical analyses were performed using SAS version 9.2 (SAS Institute, Cary, NC, USA).

### Informed Consent

Guidelines of the U.S. Dept. of Health and Human Services and the authors' institutions were followed in the conduct of this research. Approval for the HIVNET 012 study was obtained from the National HIV/AIDS Research Council in Uganda and Office of Human Subjects Research – Johns Hopkins Medicine Institutional Review Boards. Written informed consent was obtained from the women in HIVNET 012 prior to their participation in the study.

## Results

In the HIVNET 012 trial, 37 infants in the sdNVP arm and six infants in the placebo arm were HIV-infected by 6–8 weeks of age. Samples were available for analysis from 31 of those 43 infants. Table 2 shows the characteristics of the 31 HIV-infected infants included in the sub-study and the 12 infants who did not have samples available for analysis. The only difference observed was that a higher proportion of infants in the group not included in the sub-study died during the follow-up period; however, this difference was not statistically significant (P = 0.05); none of those infants died by 6–8 weeks of age, so death of those infants was not related to their lack of inclusion in the sub-study. Most of those infants had samples collected in the HIVNET 012 trial at the 6–8 week visit; the samples from HIVNET 012 were used for numerous previous studies, and those samples were used up in prior testing.

**Table 2:** Characteristics of infants who were HIV-infected by 8 weeks of age (HIVNET 012 trial, Uganda, enrollment 1997–1999).

Variable	Infants included in analysis (N = 31)	Infants not included in analysis (N = 12)	P value
Median infant CD4% at birth (range)	44.6 (20.7, 76.0)	39.3 (25.0, 52.5)	0.07^d^
Median infant log_10_ HIV viral load at 14 weeks of age (range) a	5.8 (4.7, 6.9)	6.0 (4.7, 6.9)	0.36^d^
Median maternal CD4 cell count at delivery (range)	477 (101, 1352)	379 (14.0, 1014)	0.22^d^
Median maternal log_10_ HIV viral load at delivery (range)	4.8 (3.9, 5.8)	5.0 (3.7, 5.7)	0.86^d^
Maternal HIV subtype			
A	11 (35.5%)	4 (33.3%)	0.17^e^
C	1 (3.2%)	2 (16.7%)	
D	10 (32.3%)	3 (25.0%)	
Intersubtype recombinant	3 (9.7%)	3 (25.0%)	
Unknown	6 (19.4%)	0 (0.0%)	
Single dose NVP exposure			
Exposed	25 (80.6%)	12 (100.0%)	0.16^e^
Non-Exposed	6 (19.4%)	0 (0.0%)	
Timing of infant HIV infection b			
HIV-infected *in utero*	20 (64.5%)	9 (75.0%)	0.72^e^
HIV-infected after birth by 6–8 weeks	11 (35.5%)	3 (25.0%)	
NVP resistance in infants at 6–8 weeks c			
No resistance	10 (47.6%)	3 (100%)	0.22^e^
Resistance	11 (52.4%)	0 (0.0%)	
Death (5 years)			
No	16 (51.6%)	2 (16.7%)	0.05^e^
Yes	15 (48.4%)	10 (83.3%)	
Proportion breastfeeding			
At 6 months of age	27 (87.1%)	10 (83.3%)	1.00^e^
At 12 months of age	25 (80.6%)	10 (83.3%)	1.00^e^
Initiated ART during HIVNET 012			
Yes	1 (3.2%)	0 (0.0%)	1.00^e^

a Among 43 infants in the HIVNET 012 trial who were infected at birth or by 6–8 weeks, 36 had viral load data obtained at 14 weeks of age (26 included in the analysis and 10 not included in the analysis). There was insufficient viral load data available from 6–8 weeks for meaningful statistical analysis.

b Infants with *in utero* HIV infection had a positive HIV DNA test at birth.

c For 19 infants (10 included in the analysis and 9 not included), either a sample was not available for HIV genotyping or a result was not obtained.

d Two-sided exact p-value, Wilcoxon rank sum test.

e Two-sided p-value, Fisher's exact test.

We used the HRM assay to measure HIV diversity in the *gag* and *pol* genes (Gag1, Gag2, and Pol amplicons, Table 1 and Figure 1). These three regions were selected because they are likely to be subjected to different selective pressures. An advantage of using these regions for HRM analysis is that the analysis can be performed using DNA left over from HIV genotyping (resistance testing). This is important, since infant samples are often very low in volume. Our recent studies using the HRM assay to analyze HIV diversity in adults confirms that individual genomic regions (e.g., in *gag, pol*) diversify differently in different individuals (submitted manuscript). Therefore, analysis of multiple genomic regions may provide additional information relevant to biologic correlates such as survival.

Most HIV infections in Uganda are caused by HIV subtypes A and D. As a control, we first analyzed the HRM scores of two subtype A and two subtype D plasmids (cloned HIV DNA). The HRM scores of the plasmid controls were low (for all three regions: median: 3.5, range: 3.2–3.8, Table 1). Because the plasmids are clonal, these HRM scores provide baseline values that reflect the stability of the duplexes amplified from the different genomic regions (Gag1, Gag2, Pol) and their melting characteristics, in the absence of significant genetic diversity. Plasmid HRM scores obtained for the Gag1 region were slightly higher than those obtained for the Gag2 region (mean difference = 0.38, P = 0.008) and the Pol region (mean difference = 0.35, P = 0.011). HRM results for the Gag1 and Gag2 regions were obtained for all 31 infants in the sub-study at 6–8 weeks of age; one infant did not have an HRM result for the Pol region due to amplification failure. The HRM scores of the infant samples were higher than those obtained for the plasmid controls, reflecting the natural genetic diversity of HIV viruses (Table 1; P<0.0001 for Gag1, P<0.0001 for Gag2, P<0.0001 for Pol). HIV viral loads were available for 11 of the 31 infant samples from 6–8 weeks of age that were tested with the HRM assay; those viral loads were high (see Methods), indicating that sampling error was unlikely to account for the low level of diversity measured in some infant samples; however, some infants did not have viral load data obtained in the HIVNET 012 trial, and insufficient plasma remained to perform this testing for those infants. In the infant samples, as in the plasmid controls, the HRM scores obtained for the Gag1 region were slightly higher than those obtained for the Gag2 region (mean difference = 0.50, P = 0.003) and the Pol region (mean difference = 0.75, P<0.0001). We evaluated the correlation between the 6–8 week HRM scores in the Gag1, Gag2, and Pol regions [see Figure S1]. This analysis revealed an association between the Gag1 and Gag2 regions (r = 0.47, p = 0.008) and between the Gag2 and Pol regions (r = 0.41, p<0.0001), but not between the Gag1 and Pol regions (r = 0.25, P = 0.184). However, while this analysis revealed that while the low and middle range HRM scores were tightly clustered, high HRM scores in one region were not correlated with high HRM scores in the other region. This suggests that the degree of genetic diversity in the viral populations differed from one region to another, and supports the approach of analyzing the mean of HRM scores from two or three regions of the regions analyzed.

The association between clinical and laboratory variables and HIV diversity was assessed using the individual HRM scores obtained at 6–8 weeks for the Gag1, Gag2, and Pol regions, and also mean HRM scores of the Gag1 and Gag2 regions, and mean scores of all three regions. One variable examined was exposure of infants to sdNVP near the time of birth; NVP exposure could theoretically impact the diversity of the infant's viral population, either by causing bottlenecking of the population (through selection of a limited number of resistant variants) or by increasing diversity (through selection of diverse variants with resistance mutations). We did not find an association between HRM scores (above vs. below the third quartile) and sdNVP exposure; however, the ability to detect a difference between HRM scores in sdNVP-exposed vs. sdNVP-unexposed infants may have been limited by the small number of sdNVP-unexposed infants enrolled into this cohort (N = 6). We also did not find an association between HRM scores and the other variables examined, which included infant CD4 cell count % at birth, infant HIV viral load at 14 weeks, maternal CD4 cell count at delivery, maternal HIV viral load at delivery, HIV infection *in utero*, infant NVP resistance at 6–8 weeks of age, and HIV subtype (data not shown).

**Figure 2:**
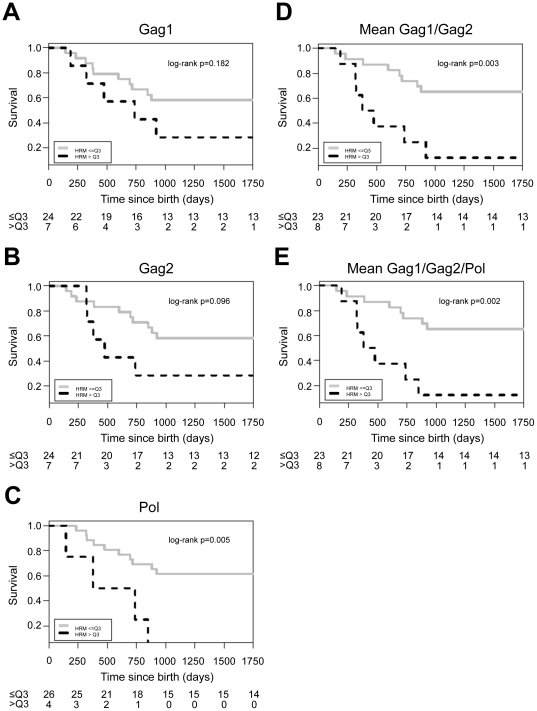
Kaplan Meier plots showing the relationship between HRM scores obtained at 6–8 weeks of age and infant survival. The figures show the Kaplan Meier analysis for survival and HRM scores. For this analysis, infants with HRM scores above the 75^th^ percentile (above the third quartile, >Q3) were characterized as having high HRM scores (black dashed line), and infants below that cutoff were characterized as having low HRM scores (grey line). The X axis shows the time since birth in days (infant age); the Y axis shows the survival probability. The number of infants still alive in each group (≤Q3, >Q3) at each time point is shown below each graph. (A) Gag1 region, (B) Gag2 region, (C) Pol region, (D) mean of the two gag regions (Gag1/Gag2), (E) mean of all three regions (Gag1/Gag2/Pol).

In this sub-study, survival was reduced among infants who had higher HRM scores at 6–8 weeks. In Kaplan Meier analyses, this association was not significant for the Gag1 or Gag2 regions individually (Figure 2A and 2B), but was significant for the Pol region (P = 0.005) and for the composite analyses (P = 0.003 for the mean of results from Gag1 and Gag2; P = 0.002 for the mean of the results from all three regions). In multivariate proportional hazard models that included HIV viral load at 14 weeks of age and HRM score, higher HRM scores at 6–8 weeks (for Gag2, the mean of results from Gag1 and Gag2, and the mean of results from all three regions) were independently associated with death (Table 3).

**Table 3:** Association of HRM score and survival in univariate (unadjusted) models and multivariate (adjusted) models that included HRM score and HIV viral load.

HRM score at 6–8 weeks	N	Hazard Ratio (95% CI) Unadjusted	P	N	Hazard Ratio (95% CI) Adjusted 1	P
Gag1	31	2.0 (0.7, 6.0)	0.19	26	2.1 (0.7, 6.6) 2	0.19
					2.6 (0.8, 9.2) 3	0.12
Gag2	31	2.5 (0.8, 7.3)	0.11	26	**3.5 (1.1, 10.9)** 2	**0.03**
					2.4 (0.7, 7.8) 3	0.15
Pol	30	4.7 (1.4, 15.8)	**0.01**	25	3.4 (1.0, 11.9) 2	0.06
					2.7 (0.7, 10.6) 3	0.15
Mean (Gag1, Gag2)	31	4.2 (1.5, 11.9)	**0.006**	26	**8.7 (2.6, 28.6)** 2	**0.0004**
					**3.4 (1.1, 10.8)** 3	**0.04**
Mean (Gag1, Gag2, Pol)	30	4.6 (1.6, 13.2)	**0.004**	26	**6.9 (2.1, 22.9)** 2	**0.002**
					1.7 (0.5, 5.3) 3	0.40

1 Two covariates were included in each multivariate model: HRM score measured at 6–8 weeks of age (binary, ≤75^th^ percentile vs. >75^th^ percentile) and HIV viral load measured at 14 weeks of age (log_10_ scale). N: number of infants included in the model; CI: confidence intervals

2 Hazard ratio, 95% CI, and P value for HRM score at 6–8 weeks of age.

3 Hazard ratio, 95% CI, and P value for HIV viral load at 14 weeks of age (log_10_ scale).

Of the 31 infants, 26 were alive at 12 months and twenty-one (81%) of the 26 infants had samples available for testing from 12 or 18 months of age (17 and 15 infants, respectively). In the analysis of longitudinal HRM scores, we found higher HRM scores were associated with older age (Table 1, beta = 0.37, P = 0.005; for Gag2: beta = 0.47, P = 0.006; for Pol: beta = 0.24, P = 0.016; where beta is the estimated mean increase in the HRM score associated with one year increase of age). In this group of infants who were infected *in utero* or shortly after birth, age is likely to serve as a proxy for time since HIV infection. Our validation studies clearly show an association between HRM scores and different, sequencing-based measures of HIV diversity, so it seems reasonable to assume that the higher scores observed in samples collected from infants at 12 and 18 months reflects mutation-driven evolution of the viral population. However, we did not sequence HIV variants from the 12- and 18-month samples.

## Discussion

Our previous study of Ugandan mother-infant pairs, which included nine infants in this study, confirmed that HRM scores are significantly associated with sequence-based measures of HIV diversity (genetic diversity, as well as complexity, and Shannon entropy) [21]. When the HRM assay is used to analyze complex molecular populations, such as DNA amplified from HIV in clinical samples, the melting temperatures of the DNA duplexes may be influenced by a variety of factors, including the number and type of nucleotide mismatches and insertions/deletions in the duplexes, as well as the proximity of those sequence differences to each other and to the ends of the duplex [22]. For those reasons, HRM scores are likely to provide a more comprehensive measure of diversity than traditional, sequenced-based approaches based on simple algorithms (e.g., the frequency of nucleotide differences in a sequence set). Both synonymous and non-synonymous mutations influence DNA melting and would be expected to influence HRM scores. Both types of mutations occur frequently in HIV, particularly in poorly conserved regions, such as HIV *gag*. The *gag* region also typically contains nucleotide insertions and deletions that would also be expected to influence HRM scores. It is important to note that many sequence-based algorithms used to measure HIV diversity ignore insertions and deletions (e.g., by “gap stripping” sequence data prior to analysis).

Melting curves can be generated using other instruments that measure incorporation/release of a fluorescent dye, such as those designed for real-time PCR. We chose to use the LightScanner instrument for the HRM assay because it was specifically designed for melt curve analysis and includes software specifically designed for high resolution melting applications. Other studies demonstrate that the LightScanner instrument has greater data density and greater temperature accuracy than other instruments [27]. Greater data density and use of the saturating LCG+ dye are key features of the LightScanner system that improve the sensitivity and accuracy of heteroduplex detection for applications such as ours that involve measuring the width of the derivative melt curve, rather than the peak melting temperature. The simplicity of the HRM assay (both in performance and data output) makes studies like this one possible. Note that this study included measurement of diversity in 31 samples collected at 6–8 weeks, 17 samples collected at 12 months, and 15 samples collected at 18 months (63 samples total), each analyzed in three different genomic regions. Furthermore, the HRM assay provides a measure of diversity in the entire viral population, which avoids the sampling bias that may be introduced with analysis of a limited number of HIV clones.

Numerous studies have evaluated changes in HIV diversity that occur during the course of HIV infection in adults [28], [29], [30], [31]. In adults, while HIV infection is usually initiated by one or a few HIV variants, some individuals are infected with multiple HIV strains [32], [33], [34], [35], [36]. When multiple HIV variants are transmitted, difference in the fitness of different HIV variants and/or selective pressures act very early in infection to select one or a few founder strains, leading to homogenization of HIV *env* sequences [37]. Antibody responses begin to contribute to genetic selection of HIV a few months after infection [38]. In the first few years of infection, *env* diversity tends to increase in a linear fashion [29], [31]. At some point, *env* diversity may stabilize or even decrease. Late in disease, *env* sequences often become homogeneous as the immune system collapses [29], [31]. While less information is available for other regions of the HIV genome, it is clear that different HIV genes/gene products are subjected to different selective pressures; for example, while *env* is the major target for anti-HIV antibodies [39], *gag* selection is mediated predominantly by cytotoxic lymphocytes (CTLs) [40]. Interestingly, the homogenization that is seen very early in infection in *env* does not appear to occur in *gag* [37]. Later in infection, HIV *env* and *gag* evolution is convergent in some individuals [40], [41].

The patterns of HIV diversification and homogenization are likely to be different in infants and adults for several reasons. First, vertical transmission results from exposure of infants to a single source: HIV from the mother. In contrast, adults often have multiple independent HIV exposures leading to HIV infection, which could influence the multiplicity of HIV infection. There are also marked differences in viral dynamics in infants and adults. In adults, there is a rapid decline in viral load shortly after HIV infection, and a viral load set point is usually established during the first few months of HIV infection. In one study, higher HIV diversity was seen in women with higher viral load set points [10]. In contrast, viral loads usually remain very high in infants during the first year of life, and then decline slowly over the next few years of HIV infection, usually remaining at levels higher than those seen in adults [42], [43]. These differences in viral dynamics may reflect the significant differences in the immune systems of newborn infants and adults. Also, infection of adults occurs in the context of a mature immune system. Antibody responses to HIV infection are typically detected in the first few weeks following infection. In contrast, the immune system in newborn infants is immature. Production of anti-HIV antibodies generally occurs later in infants, which one reason why antibody-based assays are not recommended for HIV diagnosis in children under 18 months of age [44]. Cellular responses to HIV infection are also different in children and adults. Most HIV-infected infants have deficient cytoxic T lymphocyte (CTL) responses to HIV [45], [46], and often have inadequate CD4 cell count help [42], [47]. These and other factors are likely associated with observed differences in cellular populations during HIV infection in adults vs. infants. In adults, a decline in CD4 cell count is associated with disease progression; in contrast, in children under 5 years of age, a decline in CD4 cell % is a more reliable biomarker of disease progression [48]. Furthermore, infection of infants with variants that were able to escape the mother's CTL response may further hinder the infant's ability to contain the virus [49]. For these reasons, we feel that changes in HIV diversity over time, and the relationship of HIV diversity to disease progression, are likely to be different in pediatric and adult populations. In this study, HIV diversity in infants measured using the HRM assay tended to increase during the first 12–18 months of life. We do not know if this increase in HIV diversity over time reflects direct selective pressure for evolution (e.g., a response to cytotoxic lymphocytes targeting *gag* or *pol* epitopes [50]), linkage to another region that is the target of selective pressure (e.g., antibody-induced selection of *env* epitopes), or natural accumulation of mutations in the HIV genome without selective pressure.

The major finding in this study is that higher levels of HIV diversity near the time of birth (higher HRM scores in specific regions of the HIV genome) were significantly associated with decreased infant survival. This association was seen in Kaplan Meier analyses both for the HIV *gag* region (the mean of results for Gag1 and Gag2) and the HIV *pol* region. In multivariate proportional hazard models that included HIV viral load and HRM score, higher HRM scores at 6–8 weeks (for Gag2, the mean of results for Gag1 and Gag2, and the mean of results for all three regions) were independently associated with death. Further studies are needed to examine the association between HIV diversity in other regions (e.g., HIV *env*) and infant survival.

This study differs from previous reports that examined the association between changes in HIV diversity over time in HIV-infected children and disease progression [17], [18], [19], [20]. Those studies included smaller numbers of children (five to seven in each study), used sequence-based measures for diversity analysis, analyzed the *env* region (rather than *gag* and *pol*), and focused on changes in diversity over time, rather than the level of HIV diversity early in infection. Further studies are needed to identify factors that influence the genetic diversity of HIV in young infants. In theory, maternal factors, such as high HIV viral load, high viral diversity, advanced HIV disease, or a complicated delivery, could be associated with exposure of the infant to a higher and/or more diverse viral inoculum, which could lead to establishment of infant infection with a greater number of distinct HIV variants. The genetics of the infant (e.g., HLA type, co-receptor expression) could also potentially influence the type and complexity of the viral population early in infection. If the viral population in an infant were more diverse, it might be more likely to escape immune and other selective pressures, leading to more rapid HIV disease progression. Alternatively, high levels of HIV diversity in young infants may be a surrogate marker for infection with viral variants that have more error-prone reverse transcriptase enzymes or higher rates of HIV replication; viruses with those properties might be more likely to escape immune or other selective pressures, or might cause more immune destruction over time because of increased viral replication.

We do note that the mortality in our cohort was lower than what is usually seen among HIV-infected infants in sub-Saharan Africa. In the HIVNET 012 trial (source of the samples used in this study), the 5-year mortality was 55%, similar to the mortality seen in the subset of infants analyzed in this report. The lower mortality of infants in the HIVNET 012 trial could have reflected an effect of antiretroviral drug prophylaxis or other factors, such as enrollment into a clinical trial with access to free treatment for acute illnesses, prophylaxis for other infections, immunization, and other care that may have impacted their outcome. We also recognize certain limitations of this study. These limitations include the small sample size (31 children) and the fact that 25 of the 31 infants studied were exposed to sdNVP prophylaxis, which could have influenced the composition of the viral population; in this study, we did not find an association between HIV diversity in any of the three genomic regions studied and either sdNVP exposure or detection of NVP-resistance mutations in the infant's virus.

Infants in this study were infected with non-subtype B HIV (subtypes A, C, D, and inter-subtype recombinant HIV). While we did not find an association between HIV subtype and HRM score in this study, further studies are needed to evaluate this issue. We also note that HIV viral load data was not available for some of the 6–8 week samples analyzed in the HRM assay; one or more of those samples may have had an unusually low viral load that could have resulted in a spuriously low HRM score due to sampling error. We also acknowledge that the choice of the breakpoint used in the survival analysis (above vs. below the upper quartile) was data driven. Further studies are needed to define the biologic basis for the association of HIV diversity and survival in HIV infected infants observed in this study, and to determine whether the findings of this study can be generalized.

## Supporting Information

Figure S1 **Comparison of HRM scores in the Gag1, Gag2, and Pol regions.** The plots show data from the HRM assay for the regions tested (left: Gag1 vs. Gag2; middle: Gag1 vs. Pol; right: Gag2 vs. Pol). The Pearson correlation was used to compare the HRM scores in the regions tested.
(TIF)

File S1 **Reproducibility of the HRM assay (see text).**
(DOC)

File S2 **Selection of the breakpoint (cutoff) used for analysis of the relationship between HRM scores and infant survival (see text).**
(DOC)
