# Supplementary figures and images for: Association of HIV Diversity and Survival in HIV-Infected Ugandan Infants
Source: PLoS One. 2011 Apr 14;6(4):e18642. doi: 10.1371/journal.pone.0018642 (PMC3077388; doi:10.1371/journal.pone.0018642)

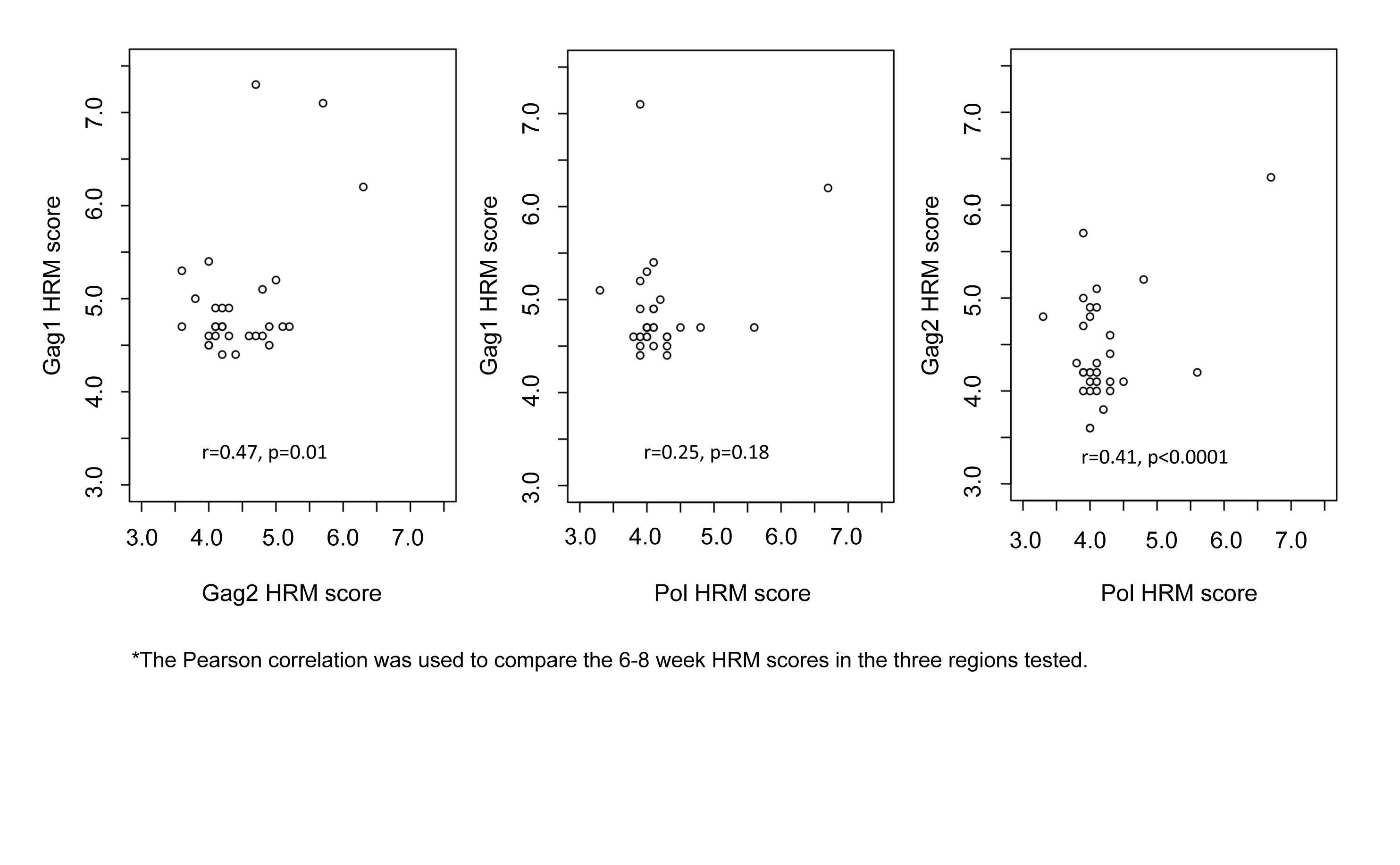

Supplement: Figure S1 — Comparison of HRM scores in the Gag1, Gag2, and Pol regions. The plots show data from the HRM assay for the regions tested (left: Gag1 vs. Gag2; middle: Gag1 vs. Pol; right: Gag2 vs. Pol). The Pearson correlation was used to compare the HRM scores in the regions tested. (TIF) [file pone.0018642.s001.tif]
