# Supplementary material for: Association of HIV Diversity and Survival in HIV-Infected Ugandan Infants
Source: PLoS One. 2011 Apr 14;6(4):e18642. doi: 10.1371/journal.pone.0018642 (PMC3077388; doi:10.1371/journal.pone.0018642)
Supplement: File S1 — Reproducibility of the HRM assay (see text). (DOC) [file pone.0018642.s002.doc]

**James et al., File S1**

**Introduction**

We recognize that many factors could influence the reproducibility of the HRM assay, including steps involved in preparation of the DNA templates, and steps involved in HRM analysis of those templates. For example, there is potential to underestimate HIV diversity when analyzing samples with lower viral loads, smaller sample volumes, or lower numbers of HIV RNA used to prepare DNA templates for HRM analysis due to sampling error (bottlenecking). There is also a concern that there may be biased amplification of HIV variants during the polymerase chain reaction (PCR) steps needed for DNA template preparation and HRM analysis. The studies below were performed to evaluate the reproducibility of the HRM assay.

**Methods**

1. Preparation of plasma samples

We obtained clinical plasma samples from three individuals with high viral loads that had GAG2 HRM scores that were typical of those seen in adults with chronic HIV infection (results for the three 500 ul samples with viral loads of 50,000 copies/ml: 7.4, 6.7, 6.7). For reference, a set of plasmids (clonal DNA, no diversity) had a median GAG2 HRM score of 3.4 (range 3.2-3.8). We then diluted the clinical samples with HIV-negative plasma to produce test samples that had viral loads ranging from 2,000 to 50,000 copies/ml. We used those test samples to prepare DNA templates for the HRM assay, using either 100 ul or 500 ul of plasma for HIV RNA extraction. A total of seven test samples were prepared for each individual (21 total test samples).

2. Preparation of DNA templates from plasma samples

The first step in preparation of DNA templates is isolation of HIV RNA from plasma. We use 1/5^th^ of the extracted RNA for reverse transcription (RT)/PCR. Using our test samples, the range of input HIV RNA used for RT/PCR ranged from 100 to 5,000 copies for each individual. The 21 extracted RNA samples were then subjected to RT/PCR in triplicate to generate DNA templates.

3. HRM analysis

The resulting 63 DNA templates were analyzed using the HRM assay (GAG2 region). Results from the RT/PCR reactions from one test sample (3 of the 63 results) were excluded from analysis for technical reasons. In a separate study, we performed HRM analysis four times over a one-year period using the same DNA templates (see below).

4. Statistical analysis

Analysis of variance was used to assess the effect of HIV viral load and plasma volume on HRM scores, and separately for the maximum input number of HIV RNA copies used for RT/PCR (a number calculated from the HIV viral load and volume used for testing). Since three RT/PCR replicates were performed for each test sample (i.e., for each sample with a given HIV viral load and volume), the replicates were treated as within-factor replication, not true replicates. Therefore, the analysis used the mean HRM score for each sample (obtained by averaging results obtained for the HRM templates prepared in triplicate) to assess differences in means. The experiment was not balanced, thus Type 3 errors were used for the analysis. All of the analyses were performed in Splus using analysis of variance.

**Results**

We found no statistically significant change in HRM scores produced by varying the viral load of the sample or the volume of the sample used for analysis (for viral load: P=0.6; for sample volume: P=0.3; F test). There was also no statistically different change in the HRM scores obtained by varying the maximum input copies of HIV RNA used for RT/PCR, even when the lowest input copy number (100 copies of HIV RNA) was compared against all other samples (P=0.6, F-test). As described above, 60 HRM results were also obtained for twenty sets of RT/PCR reactions that were performed in triplicate. The coefficient of variation for those reactions was 0.064, which represents a 6.4% variation in the HRM score in the triplicate reactions. This indicates that variability in HRM score introduced during the RT and PCR steps of template preparation is relatively low. The peak melting temperature and shape of the melting curve were very similar among the replicates for each RNA sample, providing further evidence that there was little variation in the population of viral variants amplified in replicate RT/PCR reactions used to prepare DNA templates for HRM analysis. A study evaluating the reproducibility of the nested HRM amplification step and melt curve analysis is described in a previous report (Towler et al. 2010; 26: 931-938). In that study, we assessed the reproducibility of the HRM assay (analysis of DNA templates) by analyzing 18 DNA template samples four times during a one-year period. For that analysis, the DNA templates were stored at -80^o^C. For each run, the samples were thawed and HRM amplification and data analysis were repeated. That study demonstrated that reproducibility of the HRM assay was high (intra-class correlation coefficient: 94% [95% CI: 89%, 98%]).

**Conclusions**

Results obtained with the HRM assay are not significantly affected by differences in HIV viral load (range: 2,000 to 50,000 copies/ml), sample volume (100 vs. 500 ul), or maximum number of HIV RNA copies used for DNA template preparation (range 100 to 5,000 copies of HIV RNA). DNA templates prepared using replicate RT/PCR reactions had similar HRM scores (coefficient of variation: 6.4%). There was very little variation in HRM scores obtained when the same DNA templates were analyzed repeatedly over the course of a year. These results provide support for the use of the HRM assay for analysis of HIV diversity using clinical plasma samples with variable viral loads, including low-volume infant samples.
