# Supplementary material for: Association of HIV Diversity and Survival in HIV-Infected Ugandan Infants
Source: PLoS One. 2011 Apr 14;6(4):e18642. doi: 10.1371/journal.pone.0018642 (PMC3077388; doi:10.1371/journal.pone.0018642)
Supplement: File S2 — Selection of the breakpoint (cutoff) used for analysis of the relationship between HRM scores and infant survival (see text). (DOC) [file pone.0018642.s003.doc]

**James et al., File S2**

Selection of the breakpoint (cutoff) used for analysis of the relationship between HRM scores and infant survival.

In this study, the association of HRM scores measured at 6-8 weeks of age with infant survival was assessed using two independent statistical approaches: logistic regression analysis (including univariate models and a multivariate model which, adjusted for HIV viral load, data shown in Table 3), and Kaplan-Meier analysis (data shown in Figure 2).

In both approaches, HRM data was categorized as high (above the 75^th^ percentile) or low (below the 75^th^ percentile). This choice of breakpoint (cutoff) for the continuous covariate of HRM score was data driven. Initially, two breakpoints were tested: the median value and the 75^th^ percentile. A trend for lower survival with high HRM scores was observed when the median was used as the breakpoint, but the trends were not significant. Scatter plots of the data revealed that a subset of the samples had HRM scores above the 75^th^ percentile which were associated with reduced survival. This suggested that the 75^th^ percentile was a better breakpoint to use for survival analysis.

In addition, receiver-operator-characteristics (ROC) generated using different breakpoints of HRM scores as predictors of subsequent death were examined. ROCs are more typically constructed against a “gold standard” test; here, the relative predictive strength of covariate coding choices were evaluated. Since 15 out of 16 infants remained in follow-up through the end of the 5-year follow-up period, the time-to-event outcome to the probability of death by age 5 was simplified. The binary coded HRM score at 6-8 weeks was treated as a “diagnostic test” of death by age 5 (i.e., infants with 6-8 week HRM scores greater than the breakpoint were predicted to die, while infants with 6-8 week HRM scores below the breakpoint were predicted to survive). The table below shows the “sensitivity” and the “specificity” associated with three distribution-based breakpoints: the 25^th^ percentile, the median, and the 75^th^ percentile.

Table. Sensitivity, specificity and Youden’s index (J=sensitivity+specificity-1) associated with various cutoff values of the HRM score.

| Region | Cutoff | Died | Survived | Sensitivity | Specificity | Youden’s index (J) |
| --- | --- | --- | --- | --- | --- | --- |
| Gag1 | >25^th^ percentile | 10 | 10 | 0.667 | 0.375 | 0.042 |
|  | >Median | 5 | 6 | 0.333 | 0.625 | -0.042 |
|  | **>75^th^ percentile** | **5** | **2** | **0.333** | **0.875** | **0.208** |
| Gag 2 | >25^th^ percentile | 11 | 9 | 0.733 | 0.438 | 0.171 |
|  | >Median | 8 | 7 | 0.533 | 0.563 | 0.096 |
|  | **>75^th^ percentile** | **5** | **2** | **0.333** | **0.875** | **0.208** |
| Pol | >25^th^ percentile | 9 | 13 | 0.643 | 0.188 | -0.170 |
|  | >Median | 6 | 3 | 0.429 | 0.813 | 0.241 |
|  | **>75^th^ percentile** | **4** | **0** | **0.285** | **1** | **0.285** |
| Total |  | 15 | 16 |  |  |  |

The 75^th^ percentile consistently yielded the highest Youden’s index (J), which takes into account both sensitivity and specificity; this analysis indicates that setting the breakpoint at the 75^th^ percentile better discriminates the 5-year outcome. Since the choice of the 75^th^ percentile is data driven, further independent studies would be needed to validate this association.
